# Berberine as a Potential Anticancer Agent: A Comprehensive Review

**DOI:** 10.3390/molecules26237368

**Published:** 2021-12-04

**Authors:** Abdur Rauf, Tareq Abu-Izneid, Anees Ahmed Khalil, Muhammad Imran, Zafar Ali Shah, Talha Bin Emran, Saikat Mitra, Zidan Khan, Fahad A. Alhumaydhi, Abdullah S. M. Aljohani, Ishaq Khan, Md. Mominur Rahman, Philippe Jeandet, Tanweer Aslam Gondal

**Affiliations:** 1 Department of Chemistry, University of Swabi, Anbar 23561, Pakistan; zafarali@uoswabi.edu.pk; 2 Pharmaceutical Sciences Program, College of Pharmacy, Al Ain University, Al Ain 64141, United Arab Emirates; tizneid@gmail.com; 3 University Institute of Diet and Nutritional Sciences, Faculty of Allied Health Sciences, The University of Lahore, Lahore 54000, Pakistan; aneesahmedkhalil@gmail.com (A.A.K.); mic_1661@yahoo.com (M.I.); 4 Department of Pharmacy, BGC Trust University Bangladesh, Chittagong 4381, Bangladesh; talhabmb@bgctub.ac.bd; 5 Department of Pharmacy, Faculty of Pharmacy, University of Dhaka, Dhaka 1000, Bangladesh; saikatmitradu@gmail.com; 6 Department of Pharmacy, International Islamic University Chittagong, Chittagong 4318, Bangladesh; zidankhan9090@gmail.com; 7 Department of Medical Laboratories, College of Applied Medical Sciences, Qassim University, Buraydah 52571, Saudi Arabia; f.alhumaydhi@qu.edu.sa; 8 Department of Veterinary Medicine, College of Agriculture and Veterinary Medicine, Qassim University, Buraydah 52571, Saudi Arabia; jhny@qu.edu.sa; 9 Institute of Basic Medical Sciences, Khyber Medical University, Peshawar 25100, Pakistan; ishaqkhan.ibms@kmu.edu.pk; 10 Department of Pharmacy, Faculty of Allied Health Sciences, Daffodil International University, Dhaka 1207, Bangladesh; mominur.ph@gmail.com; 11 University of Reims Champagne-Ardenne, Research Unit, Induced Resistance and Plant Bioprotection, EA 4707, USC INRAe 1488, SFR Condorcet FR CNRS 3417, Faculty of Sciences, P.O. Box 1039, CEDEX 2, 51687 Reims, France; 12 School of Exercise and Nutrition, Faculty of Health, Deakin University, Burwood, VIC 3125, Australia; tgondal@deakin.edu.au

**Keywords:** berberine, alkaloids, pharmacokinetic study, cancer preventive agents, cancer

## Abstract

Berberine (BBR), a potential bioactive agent, has remarkable health benefits. A substantial amount of research has been conducted to date to establish the anticancer potential of BBR. The present review consolidates salient information concerning the promising anticancer activity of this compound. The therapeutic efficacy of BBR has been reported in several studies regarding colon, breast, pancreatic, liver, oral, bone, cutaneous, prostate, intestine, and thyroid cancers. BBR prevents cancer cell proliferation by inducing apoptosis and controlling the cell cycle as well as autophagy. BBR also hinders tumor cell invasion and metastasis by down-regulating metastasis-related proteins. Moreover, BBR is also beneficial in the early stages of cancer development by lowering epithelial–mesenchymal transition protein expression. Despite its significance as a potentially promising drug candidate, there are currently no pure berberine preparations approved to treat specific ailments. Hence, this review highlights our current comprehensive knowledge of sources, extraction methods, pharmacokinetic, and pharmacodynamic profiles of berberine, as well as the proposed mechanisms of action associated with its anticancer potential. The information presented here will help provide a baseline for researchers, scientists, and drug developers regarding the use of berberine as a promising candidate in treating different types of cancers.

## 1. Introduction

Cancer is a disease that has become a significant public health and socio-economic concern worldwide. Hence, it seems urgent to develop strategies for the prevention and treatment of cancer [1]. Various types of cancers display resistance to chemo, radio, and hormonal therapies. Owing to these limitations, there is a dire need to develop effective, readily available, and safe anticancer therapies. Consequently, researchers are now more focused on exploring natural plant components as potential anticancer agents [2]. Plants produce numerous distinct natural products—secondary metabolites—such as terpenoids, phenolics, and alkaloids. In matrices of higher plants, phenolics and terpenoids are more abundantly present than alkaloids [3]. Among alkaloids, isoquinoline alkaloids are known as natural plant products that have demonstrated a considerable impact in drug discovery. Isoquinoline alkaloids are predominantly present in diverse plant families such as Berberidaceace, Cactaceae, Rutaceae, Fumariaceae, Papaveraceace, Magnoliaeace, Menispermaceae, Amaryllidaceae, and Ranunculaceae. These alkaloids have remarkable biological and pharmacological properties such as antifungal, anti-inflammatory, antioxidant, anticancer, antihypercholesterolemic, antidiabetic, and antimicrobial [4,5,6].

**Figure 1:**
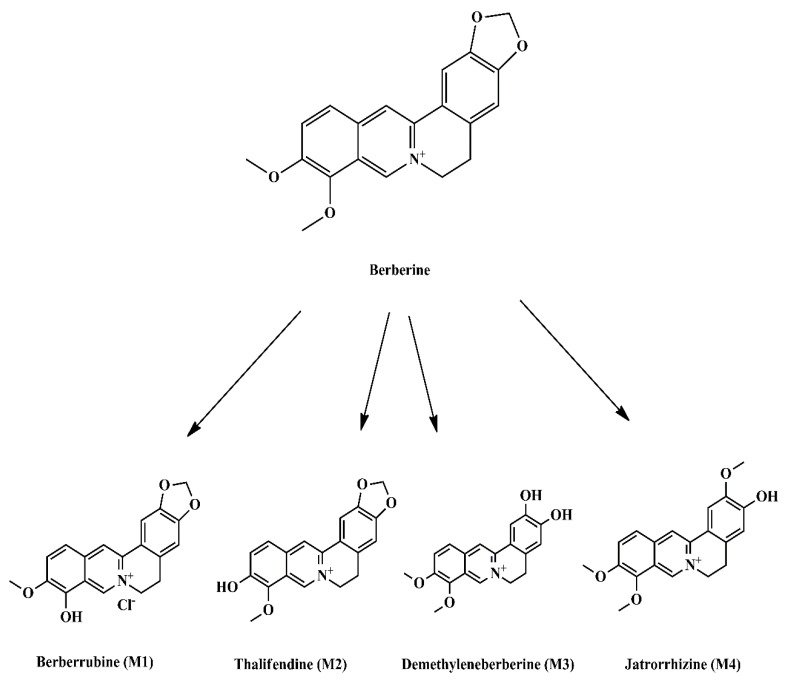
Chemical structures of berberine and its primary metabolites.

Berberine (BBR) is a benzyl tetra isoquinoline alkaloid (2,3-methylenedioxy-9,10-dimethoxyprotoberberine chloride, C_20_H_18_NO_4_^+^) (Figure 1) with a molar mass of 336.36122 g/mol. It is a well-known phytochemical compound extracted from the roots of various plants such as *Berberis vulgaris*, *B. aristotle, B. aquifolium*, *Hydrastus canadensis*, *Pellodendron chenins*, and *Coptis rhizomes* [7,8]. It is a crystal yellow-colored isoquinoline alkaloid traditionally used in Chinese and Ayurvedic medicine. Recently, scientists have reported that Berberine possesses broad-spectrum therapeutic potential due to its action against various ailments such as diabetes, hypertension, depression, obesity, inflammation, and cancer [9,10,11,12,13]. Berberine sulfate and hydrochloride have also been considered efficient herbal treatments. Scientists have reported berberine as a promising drug candidate in treating cancer [14] and various diseases such as diabetes, Alzheimer’s. It is a hydrophilic compound having low bioavailability when administrated orally; therefore, various nanotechnology-based strategies are in practice to elevate berberine bioavailability. Furthermore, coadministration with certain drugs results in increased absorption of berberine. Additionally, for decades it has served as a chemical marker in assessing the quality of various prescriptions in clinical use [15]. Therefore, this review summarizes the pharmacokinetic profile of berberine and presents an in-depth overview of its anticancer perspectives.

## 2. Sources and Extraction Techniques

Various parts (bark, stem, root, and rhizome) of plants such as goldenseal (*Hydrastis canadesis*), goldenthread (*Coptis chinesis*), barberry (*Berberis vulgaris*), Oregon grape (*Berberis aquifolium*), and tree turmeric (*Berberis aristata*) are known to contain active biomolecules such as berberine. Further, berberine has also been extracted and isolated from diverse plant genera and families, including *Tinospora* (Menispermaceae), *Annickia* (Annonaceae), *Xanthorhiza* (Ranunculaceae), *Sinopodophyllum* (Berberidaceae), *Evodia* (Rutaceae), Coelocline (Annonaceae), *Argemone* (Papaveraceae), *Rollinia* (Annonaceae), *Caulophyllum* (Berberidaceae), *Zanthoxyllum* (Rutaceae), *Xylopia* (Annonaceae), *Bocconia* (Papaveraceae), and others. Among these plants, berberine is abundantly present in several species of barberry and goldenseal that are native to America and Asia [16,17,18].

As discussed above, berberine is an alkaloid predominantly present in the matrices of various plant species, and a variety of solvents are used for its isolation. Principally, extraction methods used to isolate berberine depend on interconversion reactions among the protoberberine salt and the base itself. Apart from extraction methodologies, conversion of protoberberine salts to their specific bases is performed, and the resulting bases are further extracted using different organic solvents [19,20]. As berberine is a photo- and thermo-sensitive compound, both light and heat are considered as main challenging factors during its extraction. However, various conventional extraction methods are widely used, such as soxhlet, percolation, maceration, and continuous hot extraction, using different solvents (chloroform, ethanol, and methanol). In these conventional methods, exposure to light and heat results in the degradation of berberine, thereby reducing berberine recovery from plant matrices [21]. Currently, research is focused on employing novel and innovative extraction techniques (supercritical fluid or pressurized liquid extractions, ultrasonication, microwave-assisted extraction, and ultrahigh pressure extraction) due to their enhanced extraction efficiency, reduced extraction time, and minimal detrimental effects [22]. Choices of the solvent and the type of extraction technique are considered critical steps in both the extraction and the isolation of berberine. Table 1 gives a brief overview regarding berberine extraction using different techniques.

## 3. Pharmacokinetic Profile of BBR

In humans and mice, the primary metabolites of BBR are berberrubin (M1), thalifendine (M2), demethyleneberberine (M3), and jatrorrhizine (M4), as other alkaloids contained in the extracts of *H. candidiasis* (such as hydrastine) (Figure 1) [35,36]. The bacterial microflora of the intestine plays an important role in the enterohepatic circulation of BBR and its regulated metabolites. Recent reports have shown that the microbiota of a healthy intestinal tract helps convert berberine to its easily absorbable form, dihydroberberine, which displays a 5-fold higher intestinal absorption rate compared with its parent molecule [36]. Following administration of BBR in rats and humans, the presence of the BBR metabolites M1, M2, M3, and M4 was detected in bile, urine, and feces, as well as BBR sulfate and glucuronide conjugates [37]. The pharmacokinetic profile of BBR and its metabolites, which was extensively studied both in animal models [38] and in humans [39], demonstrated analogies between the two models, mainly regarding the low oral bioavailability of BBR, thus requiring relatively high dosages for clinical practice (0.5–1 g/month). Chen et al. studied the BBR pharmacokinetic profile in rabbits after intravenous administration of 2 mg/kg BBR sulfate, obtaining the following kinetic parameters; t_1/2(α)_: 2.32 ± 1.18 min, t_1/2(β)_: 5.28 ± 1.00 h, total plasma clearance (CL): 5.46 ± 1.62 L/h, elimination rate constant (*K*_10_): 1.75 ± 1.17 h^−1^, and an area under the concentration-time curve (AUC): 0.84 ± 0.27 μg h/mL [40].

Spinozzi et al. have reported that after a single oral intake of BBR chloride in healthy subjects (500 mg), plasmatic BBR, M3, and M4 levels (Figure 1) were very low (0.07 ± 0.01, 0.14 ± 0.01, and 0.13 ± 0.02 nM, respectively) displaying a similar pharmacokinetic profile; a plateau was reached after one hour for BBR and M3 and after 2 h for M4, persisting for up to 24 h [41]. In contrast, the plasma concentration of M1 reached 10-fold higher levels after 4 h, that is, 1.4 ± 0.3 nM, slowly decreasing to a concentration of 0.15 ± 0.02 nM after 24 h. The same authors reported that after a chronic administration of 15 mg/kg body weight/day of BBR for three months, patients with hypercholesterolemia showed plasmatic bioaccumulation of BBR and its primary metabolites. Maximum steady-state concentrations were 4.0 ± 2.0, 6.7 ± 3.0, 1.7 ± 0.3, and 5.6 ± 2.0 nM for BBR, M1, M3, and M4, respectively. Even so, M1 was the most abundant compound present in the plasma [41].

Despite the low plasmatic concentration of BBR and low bioavailability, its metabolites retained a higher concentration in the plasma, behaving as pharmacologically active forms of BBR [42,43]. Moreover, after oral administration, BBR is rapidly distributed in the body with maximum concentrations in the liver, followed by kidney, muscles, lung, brain, heart, pancreas, and fat [44].

## 4. Anticancer Perspectives

### 4.1. Breast Cancer

Triple-negative breast cancer (TNBC) is an aggressive breast cancer subtype. Berberine was cytotoxic against all treated TNBC cell lines such as MDA-MB-231, MDA-MB-468, HCC1937, HCC70, HCC38, BT-20, HCC1143, and BT-549. Among all these experimented cell lines, the most sensitive ones were HCC70 (IC_50_ = 0.19 µM), BT-20 (IC_50_ = 0.23 µM), and MDA-MB-468 (IC_50_ = 0.48 µM) [45]. Using flow cytometry techniques, BBR at 0.5 and 1 µM for 120 and 144 h not only induced cell cycle arrest at first growth (G1) and second-growth (G2)/medium phases, but it also triggered significant apoptosis [45]. Interestingly, although BBR was cytotoxic to TNBC cells, it did not affect the viability of normal human breast cells (MCF10) cultured in a 3D Matrigel model 15. These results suggest that berberine may be a suitable potential candidate for the development of a TNBC drug. Berberine addition at a dose of 1 μM to MDA-MB-468 cells induced a significant increase in the G1 phase population with a decrease in the S and G2/M phases [46].

BBR reduced the expression of the proliferating cell nuclear antigen (PCNA) protein and cyclin D1 in MDA-MB-468 cell cultures to block their progression into the G1 phase of the cell cycle. Likewise, application of BBR to MDA-MB-468 cells at a dose of 6 and 12 μM for 48 h caused a cell cycle arrest in the first growth (G1) phase with a decrease in the expression of cyclin D1 depending on the dose [46]. Zhao and Zhang recently investigated the role of berberine regarding the behavior of the MDA-MB-231 malignant breast tumor cell line. Namely, BBR reduced cell migration ability, provoked inhibition of phosphorylation, decreased overexpression of the tumor necrosis factor α (TNF-α) and Interleukin 6 (IL-6), and induced suppression of the nuclear factor kappa light chain enhancer of activated β cells (NF-Κβ) [47].

On the other hand, autophagy is a conservative mechanism for maintaining cellular homeostasis by clearing misfolded proteins and damaged organelles [48]. In cancer therapy, autophagy is seen as a double-edged sword because it can prevent early tumorigenesis and protect cancer cells later in life [49]. Thus, the combination of autophagy inhibitors and chemotherapy is expected as a promising cancer treatment strategy, and multiple autophagy inhibitors are already in the preclinical stage [50].

In MCF-7 breast cancer cells and the doxorubicin-resistant (ADR) cells MCF-7 (MCF-7/ADR), BBR was recently identified as an autophagy suppressor, inhibiting the formation of autophagosomes in MCF-7/ADR cells [51]. Berberine treatment blocked the accumulation of the LC3II protein, which is associated with autophagy, leading to accumulation of the signaling adaptor p62 protein, decreasing cell proliferation, and reversing doxorubicin resistance [52]. Mechanically, BBR inhibits autophagy by modulating the PTEN/Akt/mTOR signaling pathway. It also regulates the mitogen-activated protein kinase and the Wingless/Integrated (Wnt)/β-catenin signaling pathways in breast cancer cells [53] while suppressing chemotherapy resistance through autophagy regulation [46,54]. BBR as a potent anticancer agent significantly reduces cell viability, inhibits colony formation, cell migration, and decreases the secretion of proinflammatory cytokines (IL-1α, IL-6, TNF-α, IL-1β) [55]. BBR also increases the release of Lactic Acid Dehydrogenase (LDH) in the MDA epithelial human breast cancer cell line (MDA-cells) and downregulates the purinoceptor 7 (P2 × 7) associated with speck apoptosis, procaspase-1, and caspase-1 p20, domain recruitment (ASC), IL-1β proteins, interleukin-18 (IL-18), the mRNA expression of caspase-1 and ASC in the NOD-, and LRR- and the pyrin domain-containing protein 3 (NLRP3) inflammasome cascade [55]. Proposed mechanisms regarding the breast anticancer properties of berberine are presented in Table 2.

### 4.2. Colon Cancer

BBR treatment suppresses the viability of colorectal cancer cells by increasing their apoptosis level. The long noncoding RNA cancer susceptibility candidate 2 (CASC2) is activated in cells treated with BBR, and knockdown of the RNA CASC2 reverses BBR-induced apoptosis [56]. In addition, the antiapoptotic β-cell lymphoma-2 (*Bcl-2*) gene and *CASC2* were inhibited by treatment with berberine causing proapoptotic effects. Moreover, CASC2 lncRNA binds to the Au-rich element-binding factor 1 (AUF1), which blocks the binding of AUF1 to Bcl-2 mRNA, thereby inactivating Bcl-2 translation [56]. There are many antitumor mechanisms induced by BBR in human colorectal cancer cells, such as suppression of cell viability, induction of cell apoptosis, and upregulation of CASC2 lncRNA [56,57]. Berberine also modulates the expression of the micro-RNA-429 (MIR-429) in colorectal cancer [58]. The role of BBR in the colorectal cancer stem cells (CRC) was further explored by Liu et al. [58], who showed that this compound inhibits the invasion and metastasis of CRC cells via the prostaglandin–endoperoxide synthase 2/prostaglandin E2, mediated by the Janus kinase 2 pathway [58]. BBR inhibits the viability of CRC cell lines and promotes cell apoptosis in a dose-dependent manner. Moreover, RNA sequencing has shown that several lncRNAs may be important regulators of the BBR-dependent pathway. MiR-21 is involved in cell proliferation, invasion, invasion of blood vessels, and metastasis of many types of cancers [59]. Berberine suppresses the viability of colon cancer cells and regulates the three-gene network microRNA (miR)-21-integrin β4 (ITGβ4)—programmed cell death 4 (PDCD4) [60]. It was demonstrated that BBR treatment suppresses the viability of colon cancer cells, induces apoptosis, and activates caspase-3 activity in the human colon cancer cell line HCT116 [60]. BBR inhibits the miR-21 expression and stimulates the expression of PDCD4 proteins in the HCT116 cell line. Overexpression of miR-21 reduces the anticancer effects of BBR on cell viability, apoptosis rate, and caspase-3 activity of the HCT116 cell line [60,61]. Table 2 provides an overview of berberine action against various colon cancer cell lines and the proposed anticancer mechanisms.

### 4.3. Pancreatic Cancer

Berberine (0.3–6 μM) inhibits DNA synthesis and proliferation of pancreatic ductal adenocarcinoma (PDAC) cells and retards the development of their cell cycle in G1. BBR treatment also reduces by 70% the growth of MiaPaCa-2 cells when implanted into the flanks of nu/nu mice [62]. BBR lowers mitochondrial membrane potential and intracellular ATP levels and induces potent AMPK activation, as evidenced by phosphorylation of the AMPK α subunit at Thr172 and acetyl CoA carboxylase (ACC) at Ser79. In addition, BBR inhibits, in a dose-dependent manner, mTORC1 (phosphorylation of S6K at Thr389 and S6 at Ser240/244) and ERK activation in PDAC cells stimulated with insulin and neurotensin or fetal bovine serum [62]. Knockdown of the expression of the catalytic subunits α1 and α2 of AMPK reverses the inhibitory effect caused by the treatment with low concentrations of BBR on mTORC1, ERK, and DNA synthesis in PDAC cells. However, at higher concentrations (3 μM), BBR inhibits mitogenic signaling (mTORC1 and ERK) and DNA synthesis through an AMPK-independent mechanism [62]. Similar results were obtained with metformin used at doses that produced either a moderate or significant decrease in intracellular ATP levels, almost identical to the decrease in ATP levels observed in response to BBR [62]. One can hypothesize that BBR and metformin inhibit mitogenic signaling in PDAC cells via dose-dependent AMPK-dependent and independent pathways [63].

G-protein coupled receptors (GPCRs), and their related agonists are used as autocrine/paracrine growth factors for multiple solid tumors [64,65]. It has been shown that pancreatic cancer cell lines express multiple GPCRs [66] and various GPCR agonists, including neurotensin, angiotensin II, and bradykinin, which stimulate DNA synthesis in pancreatic cancer cell lines including PANC-1 and MiaPaca-2 [67]. In the pancreatic cancer cell lines PANC-1 and MIA-PaCa2, Park et al. [68] identified the anticancer role of berberine via a variety of pathways such as induction of phase G1. In contrast, induction of apoptosis was triggered by a mechanism involving the production of reactive oxygen species (ROS) rather than activation of caspase 3/7. Similarly, in another study, the effects of berberine and some of the modified berberines (NAX-compounds), metformin, and chemo-preventive drugs were assessed on four pancreatic adenocarcinoma cell lines (AsPC-1, BxPC-3, MIA-PaCa-2, and PANC-28). Berberine and modified berberine compounds enhanced the effects of metformin. In MIA-PaCa-2 cells, restoration of WT-TP53 activity changed the sensitivity towards metformin and modified BBRs combination compared with parent cells lacking in WT-TP53. Some modified BBRs helped alter the expression of key molecules involved in cellular growth. Therefore, the outcomes of that study concluded that combined treatment with berberines and NAX compounds may help suppress the proliferation of pancreatic cancer cells [69]. Table 2 highlights the effect of berberine against various pancreatic cancer cell lines along with its proposed mechanisms of action.

Reportedly, in human pancreatic cancer cells (BxPC-3 cells), BBR has been found to have an inhibitory action on the cellular growth of cancer cells and mediated caspase-independent cell death [70]. BBR showed inhibitory effects in pancreatic cancer cells (PANC-1, AsPC-1, and MIA-PaCa-2) on the expression of *Rad51* and the upregulation of *PARP* expression compared with control pancreatic cancer cells. The combined influence of olaparib (*PARP* inhibitor) and berberine displayed synergistic inhibitory effects on cellular activity and induced apoptotic conditions in experimented pancreatic cancer cells [71]. Based on a phenotypic assay, berberine showed a notable inhibitory role in pancreatic cancer cell metastasis and viability. Additionally, berberine treatment significantly damaged the mitochondria of pancreatic cancer cells and therefore dysregulated their energy metabolism processes [72]. In pancreatic cancer cells, BBR treatment also influenced citrate metabolism resulting in blocking of the fatty acid biosynthesis. Finally, Liu et al. [72] have proposed that BBR inhibits the proliferation of pancreatic cancer cells via the regulation of citrate metabolism and, therefore, citrate metabolism may be considered a promising target in drug development for the treatment of pancreatic cancers. Similarly, according to another study carried out in the pancreatic cancer cells PANC-1, treatment of gemcitabine (a standard drug) and BBR resulted in the reduction of side-population cells to 6.8 and 5.7%, respectively. Further, in BBR and gemcitabine-treated PANC-1 and MIA-PaCa-2 cells, all the examined stem cell-associated genes (*NOTCH1*, *NANOG*, *POU5F1,* and *SOX2*) were suppressed, except *NOTCH1*. Hence, the authors believed that the stem cell-associated genes (*NANOG*, *POU5F1,* and *SOX2*) may serve as promising markers and that BBR can be considered a potent anticancer agent for the treatment of pancreatic cancers [73].

### 4.4. Gastric Cancer

Matrix metalloproteinases (MMP) can cleave all extracellular matrix components and contribute to malignant cell invasion and metastasis. Gastric cancer has been linked to four matrix metalloproteinases (MMPs) (MMP-1, -2, -7 and -9) [74]. BBR was shown to suppress human gastric cancer cell growth and migration in a dose-dependent manner. In the gastric cancer cells SNU-5, BBR induced the production of Reactive Oxygen Species (ROS) while decreasing the nuclear factor kappa-light-chain-enhancer of activated B cells (NF-κB). BBR exerted anticancer properties in gastric cancer cells by preventing cell migration by inhibiting *MMP -1, -2,* and *-9* gene expression [75].

Pandey et al. [76] discovered that BBR impairs gastric cancer cell viability in a dose-dependent manner by inhibiting the signal transducer and activator of transcription 3 (STAT3) levels and survivin expression. These authors showed that 5-fluorouracil in combination with BBR increases gastric adenocarcinoma cell death by suppressing survivin and STAT3 expression [76]. BBR was found to suppress the activation of the epidermal growth factor receptor (EGFR) in gastric cancer tumors. Research conducted by Wang et al. [77] evaluated whether BBR could help EGFR tyrosine kinase inhibitors (TKI) function better in gastric cancer cell lines and xenograft models. They reported that BBR could effectively improve the activity of targeted standard cancer drugs such as erlotinib and cetuximab in vitro and in vivo. BBR has been shown to suppress growth and cause apoptosis in gastric cancer cell lines owing to the inhibition of EGFR signaling, which includes STAT3 phosphorylation [77]. Likewise, in gastric cancer cells (SGC7901 and AGS lines), BBR treatment suppressed cell proliferation, induced cell cycle arrest, and attenuated invasion via the down-regulation of *C-myc*, cyclin-D1, and MMP-3 expressions, respectively [78]. In reaction to BBR therapy, expression of Bcl-xL and cyclin D1 protein decreased, whereas cleavage levels of poly-ADP ribose polymerase (PARP) increased significantly [77].

In another study, the effect of BBR has also been examined in gastric cancer cells (SGC-7901 and BGC-823 lines) that were resistant to cisplatin. Purposely, coadministration of BBR and cisplatin increased the apoptotic conditions in the experimented cisplatin-resistant gastric cancer cell lines. Conclusively, it was noticed that BBR sensitized resistant cancer cells to cisplatin and increased its antigastric cancer properties owing to the inhibition of PI3K/AKT/mTOR signaling [79]. Berberine-treated gastric cancer cells (BGC-823 and SGC-7901), which were already resistant towards cisplatin, showed a reduction in cisplatin resistance due to modulatory effects on the miR-203/Bcl-w apoptotic axis and hence might increase the chemotherapeutic responses among patients having cisplatin-resistant gastric cancers [80]. In vitro and in vivo experimentations revealed the inhibitory potential of BBR in the gastric cancer cell line BGC-823 due to the induction of cytostatic autophagy through suppression of MAPK/mTOR/p70S6K and Akt signaling pathways [81]. Similarly, Li et al. [82] proposed the use of berberine hydrochloride as a potential drug candidate for the treatment of gastric cancer as this compound modulates MAPK-signaling pathways [82] (Table 2).

### 4.5. Liver Cancer

BBR inhibits cyclin D1 expression in human hepatoma cells both in vitro and in vivo in a dose- and time-dependent manner [83]. BBR allows the nuclear cyclin D1 to be released into the cytoplasm for proteasome degradation by increasing cyclin D1 phosphorylation at the Thr286 location. To foster cyclin D1 ubiquitin-proteasome-dependent proteolysis, BBR recruits skp, cullin, and the F-box containing transducing–repeat-containing protein (SCF^β-TrCP^) complex. Further, BBR blocks the turnover of cyclin D1 when β-TrCP is knocked out [83]. In hepatocellular carcinoma cells (HCC), over-expression of the Solute Carrier Family 1 Member 5 (SLC1A5) results in a poor prognosis. On the other hand, BBR has been reported to inhibit the proliferation of Hep3B and BEL-7404 cells in vitro by suppressing glutamine uptake and inhibiting SLC1A5; however, the increased activity of SLC1A5 results in an increase in glutamine uptake and an increase in BBR tolerance. In addition, BBR inhibits the growth of tumor xenografts and the expression of SLC1A5 and c-Myc in vivo [84].

BBR can cause cell cycle arrest and display anticancer properties in hepatocellular carcinoma cells (HCC). G1 step cell cycle arrest was observed in Huh-7 and HepG2 cells treated with BBR [85]. Moreover, it was found that BBR could inactivate the AKT pathway resulting in suppression of S-phase kinase-related protein 2 (Skp2) expression while it increased the expression and the nucleocytoplasmic translocation of the Forkhead box O3a (FoxO3a). On one side, translocated FoxO3a can directly promote transcription of the CDKIs p21Cip1 and p27Kip1, thus inhibiting Skp2 expression, both of which contribute to the upregulation of p21Cip1 and p27Kip1. The cell cycle is thus arrested in the HCC/G1 process [85]. BBR application was found to inhibit cell viability in the hepatocarcinoma cell lines SNU-182, Hep3B, and HepG2, due to a modulating effect on the expression of multiple tumorigenesis-related gene proteins [86]. Liver anticancer potential for BBR is mainly due to the regulation of hepatoma cells via interactions among ESR1, TB52, PTGS2, CCDN1, and MAPK1 pathways, which act on Hub-nodes in those interlinked pathways. This is related to immune–inflammatory activities such as induction of apoptotic conditions and proliferation of hepatic cancer cells [87]. According to a study conducted by Huang et al. [88], the coadministration of BBR and sorafenib synergistically inhibited the proliferation of human liver cancer cells (HepG2 and SMM-7721) in a concentration-dependent manner. Similarly, BBR minimized the cell viability of Bel-7404, HepG2, and H22 cell lines in a time-and concentration-dependent manner. Additionally, BBR significantly inhibited the expression of COX-2 (cyclooxygenase-2) and cPLA2 (cytosolic phospholipase) but increased the arachidonic acid to PGE2 (prostaglandin E2) ratio [89]. Interestingly, BBR was reported to have a selective inhibitory effect on the proliferation of hepatocellular cancer cells through induction of apoptotic conditions in AMPK-mediated caspase-dependent mitochondrial pathways through its action rarely resulted in a cytotoxic impact in normal cells [90] (Table 2).

### 4.6. Oral Cancer

BBR caused genomic DNA fragmentation, cell morphology alterations, and nuclear condensation in a dose-dependent manner in KB oral cancer cells [91]. Apoptosis and enhanced caspase-3 and -7 activities were also observed. BBR has also been shown to increase the expression of the FasL death receptor ligand [91]. As a result, the proapoptotic factors, including caspases-3, 8, and 9, as well as the poly (ADP-ribose) polymerase, were expressed. BBR also greatly improved the expression of proapoptotic factors such as Bax, Poor, and Apaf-1, Bcl-2, and Bcl-xL, while antiapoptotic factors were downregulated [91]. The activation of caspase-3 and PARP was blocked by Z-VAD-FMK, a cell-permeable pan-caspase inhibitor [91].

In athymic nude mice, BBR effectively inhibited tumorigenicity and the development of the EBV-positive NPC cell line C666-1. Successful inhibition of STAT3 activation in NPC cells within tumor xenografts grown in nude mice well correlates with the inhibition of tumorigenic development of NPC cells in vivo. BBR blocked constitutive and IL-6-induced STAT3 activation [92], which resulted in growth inhibition and apoptosis in NPC cells. IL-6 was found to be secreted by tumor-associated fibroblasts, and conditioned media from fibroblasts activated STAT3 in NPC cells [92]. BBR or antibodies to IL-6 and IL-6R may also inhibit STAT3 activation by regulatory media of tumor-associated fibroblasts [93]. Treatment with BBR impaired the development of the human esophageal squamous cell carcinoma cell line KYSE-70 and the esophageal adenocarcinoma line SKGT4 in a dose- and time-dependent manner. The inhibitory function of BBR was more sensitive in KYSE-70 cells than in SKGT4 cells. The number of cells in the G2/M process (25.94%/5.01%) was higher in KYSE-70 cells treated with 50 μmol/L BBR for 48 h than in the control (9.77%/1.28%). At 12 and 24 h after treatment, flow cytometric analysis indicated that BBR significantly increases the KYSE-70 apoptosis population relative to control cells (0.83% vs. 43.78%, 12 h) [94]. The apoptosis effect of BBR was higher at 24 h compared to 12 h (81.86% vs. 43.78% *p*%, *p* < 0.01). BBR blocked the phosphorylation of rapamycin and Akt, the mammalian targets of P70-S6-Kinase, and increased AMP-activated protein kinase phosphorylation in a prolonged fashion, according to Western blotting [94] (Table 2).

### 4.7. Bone Cancer

In vitro and in vivo administration of BBR to osteosarcoma cells reduces the expression of caspase-1 and Interleukin-1 (IL-1) in tumor cells and inhibits tumor cell development. It was suggested for the first time that BBR inhibits the caspase-1/IL-1 inflammatory signaling axis, resulting in antiosteosarcoma properties [95]. BBR has a possible genotoxic effect on human osteosarcoma cells, as determined by DNA fragmentation analysis and flow cytometry, by dramatically increasing apoptosis in a concentration and time-dependent manner [96]. In the osteosarcoma U-2 OS cells, BBR and BBR nanoparticles made of heparin (HP), reduced cell viability, arrested the cell cycle in the G1 phase, and reduced expression of the mouse 2 min 2 homologs (MDM2) [97]. The PI3K/Akt pathway was activated, rising *Bcl-2* (B-cell lymphoma 2) expression. BBR prevents PI3K/AKT activation resulting in an increased expression of *Bax* (Bcl-2-associated X protein) and *PARP* (Poly (ADP-ribose) polymerase) and decreased expression of *Bcl-2* and *caspase*-3 [97]. Overall, BBR inhibits the activation of the PI3K/Akt signaling pathway, which hinders human osteosarcoma U2OS cell proliferation and induces apoptosis [97]. BBR inhibits human chondrosarcoma cell migration and invasion by downregulating v3 integrins via the protein kinase C (PKC) and the proto-oncogene tyrosine-protein kinase, c-Src [98].

BBR (40−160 μmol/L) inhibits cell proliferation and IL-6 secretion in U-266 (human, peripheral blood, multiple myeloma) cells in a time and dosage-dependent manner. BBR, on the other hand, decreases miR-21 and Bcl-2 levels and induces ROS formation, G2/M step arrest, and apoptosis in U266 cells [99]. BBR-induced inhibition of cell proliferation and IL-6 secretion was disrupted by overexpression of miR-21. The activity of NF-κB was reduced by around 50% in U266 cells treated with BBR (80 μmol/L), followed by a substantial decrease in miR-21 levels. BBR (80–160 μmol/L) increases Set9 (lysine methyltransferase) levels by more than two-fold, resulting in methylation of the RelA subunit, which in turn inhibits NF-κB nuclear translocation and miR-21 transcription. In U266 cells treated with BBR (80 μmol/L), knocking down Set9 with siRNA resulted in a substantial rise in NF-κB protein levels and a partial recovery of cell proliferation. BBR prevents multiple myeloma development by downregulating three miRNA clusters and a significant number of mRNAs via the TP53, Erb, and MAPK signaling pathways. The mir-99a to 125b cluster may be a potential therapeutic target for multiple myeloma [100].

**Figure 2:**
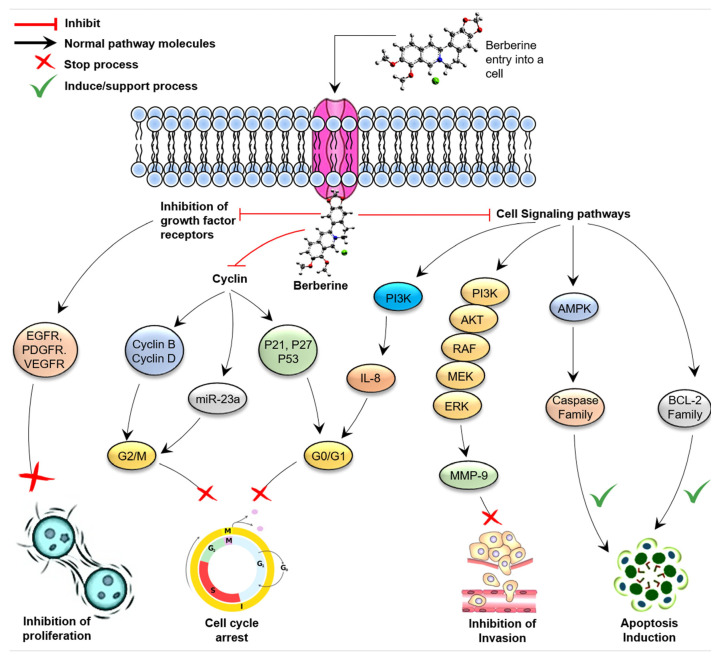
Anticancer mechanisms of berberine.

IL-6 regulates miR-21 transcription in IL-6-dependent human myeloma cell lines (HMCL) through signal transducers and activators of transcription 3 (STAT3)-related mechanisms [101]. Importantly, in the absence of IL-6, the ectopic expression of miR-21 is necessary to maintain the development of IL-6-dependent MM cells. As expected, the tumor suppressor programmed cell death 4 (PDCD4) is a miR-21 target. MiR-21 regulates PDCD4 directly, according to luciferase reporter review assays. Signal transducers and transcription activators 3 will target the miR-21 promoter according to bioinformatics analysis (STAT3); BBR can inhibit miR-21 transcription in multiple myeloma by downregulating IL-6 through STAT3 downregulation. Apoptosis, G2 step cell cycle arrest, and colony suppression were also caused by BBR and seed-targeting anti-miR-21 oligonucleotides in multiple myeloma cell lines (Table 2). Short interfering RNA depletion of PDCD4 could preserve BBR-induced cytotoxicity in multiple myeloma cells [102]. The anticancer mechanisms of berberine are presented in Figure 2.

### 4.8. Cancer of the Glioblastoma

BBR-mediated apoptosis blocks the AMPK/mTOR/ULK1 pathway and decreases tumor growth in glioblastoma polymorphic (GBM) cells in vivo [103]. The glioma microenvironment is characterized by inflammation. IL-1 and other neuroinflammatory cytokines secreted by glioma cells are believed to play a role in tumor initiation and progression [104]. Inflammatory responses and cancer are linked by certain intrinsic pathways, which induce cancer-causing genetic changes, with IL-1 playing a key role in these mechanisms. IL-1, for example, is a downstream effector of Ras activation and NF-κB regulatory gene activation, which is necessary to provide a favorable microenvironment for tumor formation [105]. A recent second phase of a clinical trial of a recombinant IL-1R antagonist for multiple myeloma has shown a favorable safety profile and reduced morbidity, demonstrating that anti-IL-1 therapy is a viable cancer treatment option [106]. BBR inhibits the inflammatory cytokine caspase-1 activation through ERK1/2 signaling as well as glioma cells’ subsequent development of IL-1 and IL-18. BBR therapy also decreases motility and induces apoptosis in U251 and U87 cells [107]. Furthermore, BBR has the potential to reverse the mechanism of epithelial–mesenchymal metastasis, which is a sign of tumor invasion [107].

BBR inhibits tumor development by regulating the differentiation and the role of stem cells and inducing cell death in neuroblastoma cells. Around the same time, inhibiting the adrenergic signal slows neuroblastoma development and increases cell differentiation. Calvani et al. [108] have summarized the potential benefits of BBR in inhibiting tumor growth and development in different types of cancer, especially neuroblastoma [108], BBR (6.25–200 μmol/L, 6–48 h) impaired cell viability and proliferation of U87 and U251 human glioblastoma cell lines in BALB/c nude mice (IC_50_ of 42 and 32 μmol/L, respectively). BBR (50 μmol/L) prevented HUVEC cell migration in the transwell assay by 67.50 ± 8.14% and the Matrigel assay by 73.00 ± 1.12% [109]. In the ectopic xenograft form, BBR (50 mg/kg) greatly decreased tumor weight (401.2 71.5 mg vs. 860.7 117.1 mg in the vehicle group) [109]. The hemoglobin content was greatly decreased by BBR (28.81 ± 3.64 μg/mg vs. 40.84  ±  5.15  μg/mg in the vehicle group, *p*  <  0.001). BBR (50 mg/kg) greatly increased the survival rate of mice in a stereotactic xenograft model. BBR inhibited VEGFR2 and ERK phosphorylation [109] (Table 2).

### 4.9. Skin Cancer

Various studies have revealed the anticancer role of BBR via inhibition of cell migration and invasion in different human cancer cells. Likewise, BBR administration (0–2 μM) resulted in an induction of cellular morphological alterations and decreased the number of viable cells in human melanoma skin cancer cells (A375.S2 and A375.S2/PLX resistant cells). Furthermore, BBR suppressed the migration and invasion of the melanoma skin cancer cells A375.S2. Post 24-h treatments with BBR in A375.S2 cells led to an inhibition of *SOS-1*, *p-AKT*, *MMP-1*, *NF-κB*, *Ras*, *p-FAK,* and *MMP-13* gene expression and an increase in the levels of PI3K and PKC [110]. BBR was found earlier to suppress the proliferation of skin squamous carcinoma cells (A431) in a time- and concentration-dependent manner. Moreover, BBR treatment induced different biochemical changes, such as loss of the membrane potential of mitochondria, cytochrome-c release into the cytosol, and cleavage of the poly (ADP) ribose polymerase. Results revealed that BBR induces apoptotic conditions and inhibits skin squamous carcinoma cells [111].

Similarly, Kou et al. demonstrated that BBR decreases the migration and invasion of melanoma cells B16 cells and diminishes the expression levels of RARα (retinoic acid receptor-α), p-AKT, and p-PI3K while upregulating the expression levels of RARβ (retinoic acid receptor-β) and RARγ (retinoic acid receptor-γ). The authors were of the view that in mouse melanoma B16 cells, BBR reversed the epithelial to mesenchymal transition and hence can be used as an effective anticancer agent in treating melanoma via regulation of the PI3K/Akt pathway [112]. Another group of researchers also studied the combined effect of berberine with doxorubicin on murine melanoma B16F10 cells both in vitro and in vivo. The combined treatment revealed strong inhibitory effects on cell growth and induced cell cycle (G2/M) arrest along with the reduction in Kip1/p27. Further, compared with the control, combined BBR and doxorubicin treatment caused a reduction in tumor weight (78%) and volume (85%) in B16F10 xenograft. Therefore, the authors suggested the usage of BBR and doxorubicin as a potent combination for the inhibition of melanoma cancer cell growth [113]. In melanoma A375 cells, treatment with BBR was also found to decrease the metastatic potential of cancer cells due to AMPK activation and inhibition of the ERK-signaling pathway, while the levels of COX-2 proteins were also reduced [114] (Table 2).

### 4.10. Uterus or Endometrium Cancer

Among various gynecological malignancies, endometrial cancer (EC) is recognized as the third most malignant after breast and cervical cancers [115]. Berberine has been reported to be an effective natural alkaloid having antiendometrial cancer properties. According to the in vitro and in vivo studies conducted by Wang and Zhang [116], BBR inhibited proliferation, migration, and invasion as well as metastasis in endometrial cancers. They further reported that BBR inhibits cancer cells via COX-2/PGE2-signaling pathways. In endometrial cancer cells, modulation of COX-2 was achieved as berberine activated the transcription of miR-101 through AP-1 (activator protein-1). Conclusively, BBR may be a promising candidate in treating EC as it inhibits cancer cells through miR-101/COX-2/PGE2-signaling pathways [116]. In EC cells, BBR affects the distribution of the cell cycle and induces apoptotic conditions via activation of the mitochondrial-caspase pathway. Furthermore, since BBR engaged the PI3K/Akt pathway, it may be recommended as a functional ingredient for the prevention and treatment of endometrial cancers [117].

### 4.11. Prostate Cancer

Hypoxia and ionizing radiations (IR) were used to treat the prostate cancer cell lines LNCaP and DU-145 with or without BBR therapy [118]. LNCaP cells were also xenografted into naked mice and treated with IR or BBR. BBR improved the radiation sensitivity of prostate cancer cells and xenografts in a dose-dependent manner, which was linked to inhibition of the expression of HIF-1 and VEGF [118]. BBR suppressed proliferation of the human prostate carcinoma epithelial cell line 22Rv1 and decreased cellular testosterone synthesis in a dose-dependent manner [119]. BBR inhibited the activity of the C3 enzyme from the Aldo-keto reductase family 1, rather than affecting mRNA or protein expression [107]. BBR will thus join the active core of aldo-keto reductase family 1 member C3 and form an association with the amino acid residues Phe306 and Phe311, according to molecular docking studies. Finally, the association of BBR with the aldo-keto reductase family 1 member C3 inhibits 22Rv1 prostate cancer cell development by inhibiting this enzyme and by reducing intracellular androgen synthesis [119].

In addition, BBR inhibited the androgen receptor (AR) transcriptional function in castration-resistant prostate cancers (CRPC). BBR has little effect on the expression of AR mRNA but causes AR protein degradation. Several ligand-binding domains truncated AR splice variants have been discovered, and these variants are thought to help patients develop CRPC. Surprisingly, these variants were found to be more vulnerable to BBR-induced degradation than full-length AR. BBR also impairs the development of LNCaP xenografts in nude mice and decreases AR expression in tumors, while normal prostate morphology and AR expression are unaffected [120]. BBR has been shown to suppress the capacity of prostate cancer cells to spread and infiltrate, these cells being strongly metastatic [49]. The inhibitory activity of BBR resulted in a substantial reduction in the expression of a panel of mesenchymal genes that control developmental EMT. High *BMP7*, *NODAL,* and *Snail* gene expression in metastatic prostate cancer tissues is associated with shorter survival in patients with prostate cancers and offers potential therapeutic targets among the EMT-related genes downregulated by BBR [49] (Table 2).

### 4.12. Thyroid Cancer

BBR inhibited RET expression in medullary thyroid carcinoma (MTC) cells by more than 90% at a concentration of 2.5 µg/mL but did not affect TPC1 cells [121]. Canadine, a structural analog of BBR, did not affect RET expression in MTC TT cells and had little interaction with the RET G-quadruplex. In TT cells, the BBR-mediated downregulation of RET inhibits cell proliferation by causing cell cycle arrest and activation of apoptosis, as evidenced by a two-fold increase in caspase-3 activity and downregulation of cell cycle regulation [122]. Two thyroid cancer cell lines, 8505C and TPC1, exhibited a dose-dependent growth decrease upon BBR treatment. Following BBR treatment, 8505C cells displayed a significant increase in apoptosis, whereas TPC1 cells showed cell cycle arrest at the G0/G1 phase [123]. After BBR therapy, immunoblots of p-27 expression revealed BBR caused a mild upregulation of p-27 in 8505c cells but a moderate upregulation of p-27 in TPC1 cells [123].

## 5. Conclusions

Cancer is a large category of disease that severely affects people’s health. Thus, there is a vital need for cancer prevention and treatment advancement. Surgery, radiotherapy, and chemotherapy are the most often used approaches to cancer care. People may also abandon anticancer treatments due to their ineffectiveness and adverse side effects, resulting in the illness progression and reduced overall survival rate. Resistance to anticancer drugs may be conferred by target alteration, drug-efflux pumps, increased cellular tolerance to apoptosis, increased DNA harm tolerance to therapy, reparability, and enhanced neoplastic proliferation. Resistance may be due to improvements in the stroma and tumor climate as well as cancer microenvironments. Cancer cells utilize a number of these pathways, complicating clinical strategies for each patient. Recent advancements in cancer care, such as selective and immunotherapy, also provided substantial benefits.

However, during the last decade, several clinical studies and lab analyses have been conducted to investigate the efficacy of BBR in curing cancer. Additionally, BBR was found to control pro and anticancer miRNAs and lncRNA levels and has been shown to improve the effectiveness of chemotherapy and radiation therapy. However, BBR’s direct cytotoxic impact is not considered very powerful. It acts at concentrations sometimes greater than 100 µM for certain cancer cell lines. Nevertheless, the cytotoxic action of BBR is moderate as it ranges from 10 to 100 μM. BBR’s slow absorption, efflux from intestinal cells by P-gc, and comprehensive metabolism by intestinal and hepatic cells render difficult its use in vivo. Consequently, progresses must be made on developing both the pharmacokinetic profile and the anticancer efficacy of BBR in the future. As BBR shows promising efficacy concerning anticancer potential, it may be a potential candidate in innovative anticancer drug discovery.

## Figures and Tables

**Table 1:** Overview of various techniques for berberine extraction.

Source	Plant Part	Extraction Method(s)	References
*Berberis aristata*	Roots	Microwave-assisted subcritical water extraction	[23]
*Coscinium fenestratum*	Stems	Sonication	[24]
*Berberis lyceum*	Roots	Soxhlet extraction	[25]
*Coscinium fenestratum*	Stems	Hot and cold extraction	[26]
*Berberis aristata*	Stem bark	Hot extraction	[27]
*Berberis integerrima*	Stems, leaves, and fruits	Maceration and pulsed electric field assisted extraction	[28]
*Coptis chinensis*	Rhizome	Supercritical fluid extraction	[29]
*Phellodendri amurensis cortex*	Barks	Ultrahigh pressure extraction, ultrasonic extraction, soxhlet extraction, heat reflux extraction	[30]
*Berberis tinctoria*	Stem bark	Hot extraction	[27]
*Berberis thunbergii*	Stems, leaves and fruits	Maceration and pulsed electric field assisted extraction	[28]
*Phellodendri amurensis cortex*	Barks	Ultrasound-assisted extraction	[31]
*Hydrastis canadensis*	Roots	Pressurized hot water extraction, reflux extraction, ultrasonication	[32]
*Tinospora cordifolia*	Stems	Microwave-assisted extraction, soxhlet extraction, maceration	[33]
*Mahonia manipurensis*	Stem bark	Cold extraction	[34]

**Table 2:** Summarized data of berberine effects against various cancers and their proposed mechanisms.

Cancer Type	Experimental Model (s)	Dose	Proposed Mechanism (s)	References
Breast Cancer	MDA-MB-231, MDA-MB-468, HCC1937, HCC70, HCC38, BT-20, HCC1143 and BT-549	0.2, 0.5 and 1.0 µM	Induction of G1 and -G2/M phase cell cycle arrest, Stimulation of apoptosis in cancer cells	[45]
	MDA-MB-468	6 and 12 μM	Cell cycle arrest at G1 phase, Decrease in cyclin D1 expression	[46]
	MDA-MB-231	25 μM/L	Reduction of cell migration, Phosphorylation inhibition, Decrease of TNF-α and IL-6 overexpression	[47]
	MCF-7/ADR	100 μM	Inhibition of the formation of autophagosomes	[51]
	MCF-7/ADR	100 μM	Blocking the accumulation of the LC3II protein, Decrease of cell proliferation, Reversion of doxorubicin resistance	[52]
	MDA-MB-231	2.5–100 μg/mL	Reduction of cell viability, Inhibition of colony formation, cell migration, and decrease of the secretion of the proinflammatory cytokines (IL-1α, IL-6, TNF-α, IL-1β)	[55]
Colon cancer	HT29, HCT116	0–100 μM	Upregulation of LncRNA CASC2, Suppression of Bcl-2 gene	[56]
	HCT116	1, 10 or 100 µM	Induction of apoptosis, Promotion of caspase-3 activity	[60]
Pancreatic cancer	PANC-1, MiaPaCa-2	0.3–6 µM	Inhibition of DNA synthesisCell cycle arrest at G1	[62]
	PANC-1, MiaPaCa-2	15 µM and 10 µM	Cell cycle arrest at G1, Induction of apoptosis	[68]
	AsPC-1, BxPC-3, MIA-PaCa-2 and PANC-28).	100, 1000 and 10,000 nM	Suppression of the proliferation of cancer cells	[69]
	BxPC-3	10–200 µM	Mediation of caspase-independent cell death	[70]
	PANC-1, MiaPaCa-2, AsPC-1	5 µM	Induction of apoptosis, Inhibition of *PARP* and *Rad51* expression	[71]
	PANC-1	2.5, 3.75, 5 and 10 μM	Damage of the mitochondria of pancreatic cancer cells, Targeting citrate metabolism	[72]
	PANC-1, MiaPaCa-2	10 µM, 15 µM	Downregulation of *NANOG, POU5F1,* and *SOX2*	[73]
Gastric cancer	SNU-5	75 µM	Inhibition of *MMP-1, -2* and *-9* gene expression	[75]
	AGS	0–50 µM	Suppression of survivin and STAT3 expression	[76]
	SGC7901, MKN45, BGC823	15–90 µM	Downregulation of the expression of Bcl-xL and cyclin-D1 proteins	[77]
	SGC7901, AGS	10–80 μM	Cell cycle arrest, Attenuation of tumor invasion via the down-regulation of *C-myc*, cyclin-D1, and MMP-3 expressions	[78]
	BGC-823, SGC-7901	1–1000 μM	Inhibition of PI3K/AKT/mTOR signaling	[79]
	BGC-823, SGC-7901	10 μM	Modulation of the miR-203/Bcl-w apoptotic axis	[80]
	MGC 803	0–60 μM	Modulation of MAPK-signaling pathways	[82]
Liver cancer	HepG2	0, 50 and 100 µM	Inhibition of cyclin D1 expression	[83]
	Hep3B, BEL-7404	50–125 μM	Suppression of glutamine uptake, Inhibition of SLC1A5	[84]
	HepG2, Huh-7	30–120 μM	Induction of G1 phase cell cycle arrest in cancer cells	[85]
	SNU-182, Hep3B, HepG2	10–100 μM	Modulation of the expression of multiple tumorigenesis-related gene proteins	[86]
Oral cancer	KB	0, 0.1 and 1 μg/mL	Induction of apoptosis, Enhancement of caspase-3 and -7 activities	[91]
	C666-1, HONE1, & HK1	0–50 μM	Inhibition of STAT3 activation	[92]
	HONE1	0–300 μM	Inhibition of STAT3 activation	[93]
Bone Cancer	Saos-2, MG-63	0–100 μM	Inhibition of the caspase-1/IL-1 inflammatory signaling axis	[95]
	MG-63	0–80 μM	Induction of apoptosis in cancer cells	[96]
Glioblastoma cancer	U251, U87	100 μM	Induction of autophagy	[103]
	U251, U87	50 μM, 100 μM	Inhibition of inflammatory cytokine caspase-1 activation	[107]
Skin cancer	A375.S2	0–2 μM	Inhibition of MMP1, MMP13, uPA, and Ras expressions	[110]
	A431	0–100 μg/mL	Inhibition of cancer cell proliferation, Induction of apoptosis	[111]
	B16	5–160 μM	Down-regulation of p-PI3K, p-AKT expressions, Up-regulation of RARβ and RARγ expressions	[112]
Prostate cancer	LNCaP, DU-145	20–400 μM	Inhibition of VEGF and HIF-1α expressions	[118]
	LNCaP, 22Rv1, PC3M, PC3	12.5–50 μM/L	Decrease of cellular testosterone synthesis in a dose-dependent manner	[119]
	LNCaP, 22Rv1, PC3	0–100 μM	Suppression of androgen receptor signaling	[120]

## Data Availability

Available data are presented in the manuscript.
